# Evolutionary adaptations of biofilms infecting cystic fibrosis lungs promote mechanical toughness by adjusting polysaccharide production

**DOI:** 10.1038/s41522-016-0007-9

**Published:** 2017-01-23

**Authors:** Kristin Kovach, Megan Davis-Fields, Yasuhiko Irie, Kanishk Jain, Shashvat Doorwar, Katherine Vuong, Numa Dhamani, Kishore Mohanty, Ahmed Touhami, Vernita D Gordon

**Affiliations:** 1 Department of Physics and Center for Nonlinear Dynamics, The University of Texas at Austin, 2515 Speedway, Stop C1610, Austin, TX USA; 2 Department of Molecular Biosciences, The University of Texas at Austin, NMS 3.316, Stop A5000, 2506 Speedway, Austin, TX USA; 3 Department of Biology and Biochemistry, The University of Bath, Claverton Down, Bath, UK; 4 Department of Molecular Biology, Umeå University, Umeå, Sweden; 5 Department of Petroleum and Geosystems Engineering, The University of Texas at Austin, 200 E. Dean Keeton St., Stop C0300, Austin, TX USA; 6 Department of Physics and Astronomy, The University of Texas Rio Grande Valley, One West University Blvd., Brownsville, TX USA; 7 Institute for Cellular and Molecular Biology, The University of Texas at Austin, MBB 1.220, 2500 Speedway, A4800, Austin, TX USA

## Abstract

Biofilms are communities of microbes embedded in a matrix of extracellular polymeric substances, largely polysaccharides. Multiple types of extracellular polymeric substances can be produced by a single bacterial strain. The distinct polymer components of biofilms are known to provide chemical protection, but little is known about how distinct extracellular polysaccharides may also protect biofilms against mechanical stresses such as shear or phagocytic engulfment. Decades-long infections of *Pseudomonas. aeruginosa* biofilms in the lungs of cystic fibrosis patients are natural models for studies of biofilm fitness under pressure from antibiotics and the immune system. In cystic fibrosis infections, production of the extracellular polysaccharide alginate has long been known to increase with time and to chemically protect biofilms. More recently, it is being recognized that chronic cystic fibrosis infections also evolve to increase production of another extracellular polysaccharide, Psl; much less is known about Psl’s protective benefits to biofilms. We use oscillatory bulk rheology, on biofilms grown from longitudinal clinical isolates and from genetically-manipulated lab strains, to show that increased Psl stiffens biofilms and increases biofilm toughness, which is the energy cost to cause the biofilm to yield mechanically. Further, atomic force microscopy measurements reveal greater intercellular cohesion for higher Psl expression. Of the three types of extracellular polysaccharides produced by *P. aeruginosa*, only Psl increases the stiffness. Stiffening by Psl requires CdrA, a protein that binds to mannose groups on Psl and is a likely cross-linker for the Psl components of the biofilm matrix. We compare the elastic moduli of biofilms to the estimated stresses exerted by neutrophils during phagocytosis, and infer that increased Psl could confer a mechanical protection against phagocytic clearance.

## Evolution: Toughening up biofilms in lungs

Bacteria in lungs of people with cystic fibrosis can evolve through decades to build a tough biofilm that resists the body’s defences. Vernita Gordon and colleagues at the University of Texas, with co-workers in Europe, examined biofilms cultured from lung samples taken from patients at intervals over many years. The infecting bacterial populations had steadily evolved to increase production of specific carbohydrate components of the biofilms. The researchers found that increasing production of one carbohydrate component strengthens the biofilms, most likely due to the carbohydrate being crosslinked by protein molecules. The investigation suggests that the mechanics of the carbohydrate-protein network protects the biofilms from being broken into smaller pieces that can be engulfed by defensive cells called phagocytes. This new insight into the evolution of mechanical toughness complements the previously observed evolution of increasing chemical protection. Understanding these processes will assist efforts to combat them.

## Introduction

Annually, biofilm infections affect 17 million Americans, cause at least 550,000 American deaths, and cost the US healthcare system billions of dollars.^1–3^ Biofilms are communities of bacteria that are embedded in a matrix of extracellular polymers (EPS) largely composed of bacteria-produced polysaccharides. Mechanical removal is often required to clear biofilm infections,^4–6^ because they resist antibiotics as well as other antimicrobials and evade the host immune defense.^4–8^ The mechanical integrity of the biofilm matrix leads to antibiotic resistance since the stable spatial arrangement of bacteria gives rise to differentiated microenvironments with phenotypic antibiotic resistance;^9^ indeed, mechanical breakup of biofilms can render bacteria more susceptible to antibiotics.^10^

One well-studied and pernicious bacterial species is *Pseudomonas aeruginosa*, which forms biofilm infections in the lungs of patients with cystic fibrosis (CF); these infections often last for decades. The matrices of *P. aeruginosa* feature three known polysaccharides: Psl, Pel, and alginate. In CF infections, production of alginate is well-known to increase over time;^11, 12^ more alginate is associated with poorer outcomes for patients because alginate chemically protects the biofilms.^13, 14^ It has recently been found that CF infections also evolve to increase the production of Psl.^15–17^ Psl is also known to protect biofilms from antibiotics, by chemical binding, and from the immune system, by an unknown mechanism.^18, 19^

Decades-long infections of *P. aeruginosa* biofilms in the lungs of CF patients are convenient natural models for what biofilm characteristics contribute to fitness. However, the importance of *P. aeruginosa* biofilm infections extends well beyond CF. Patients with diabetes are particularly vulnerable to the development of chronic wounds, which can cost, per patient, tens of thousands of dollars per year.^2^ Chronic wounds are characterized by a lack of healing, which largely results from infection by bacterial biofilms dominated by *P. aeruginosa* and *Staphylococcus aureus* ^5^—these can even lead to amputation. In patients with chronic obstructive pulmonary disease, *P. aeruginosa* acquisition is associated with exacerbation and can lead to long-term infection.^20^ In these and in additional cases of *P. aeruginosa* biofilm infection, the biofilm is able to resist immunological clearance. It has long been appreciated that biofilm matrix materials can provide chemical protection against clearance. The role of *mechanical* protection has been appreciated much less and has not been characterized in depth.

Our studies employ biofilms grown from clinical bacterial isolates from CF patients and biofilms grown from genetically-manipulated lab strains. Here, we show for the first time that changes in polysaccharide production can increase the mechanical toughness of biofilms, which is equivalent to increasing the energy cost of overcoming the material integrity of the biofilm. Furthermore, different EPS materials accomplish this in different ways. Increased Pel or alginate increase the yield strain of the biofilm; yield strain measures how far the biofilm can be deformed before mechanical failure, or yielding, begins. In contrast, Psl increases the elastic modulus, so that more energy is required *per* unit deformation. Psl increases the elastic modulus only when it is co-produced with CdrA, a protein that binds to Psl.^21^

We use two techniques to explore the mechanical properties of our biofilms. The first tool is a bulk rheometer, which provides the standard way to characterize complex fluids. The second tool is atomic force microscopy (AFM), which lets us measure directly the energy required to pull two bacteria apart. We find that increased Psl results in a greater energy cost expended to separate two bacteria. Energy of de-cohesion increases because increased Psl production increases both the distance over which the inter-bacterial cohesion force is exerted and the maximum value of that force, and thus the mechanical work of separation. Increasing cohesion lengthscale is directly analogous to the tactics used to design greater toughness into engineering materials.^22^ These experiments provide the first direct measurements of inter-bacterial cohesion.

## Results

Previous measurements of *P. aeruginosa* biofilm moduli have reported ranges of values that vary over orders of magnitude (from a few Pa to tens or hundreds of kPa).^23–30^ It is not clear to what degree this reflects differences between bacterial strains vs. differences resulting from measurement techniques or culturing conditions. To circumvent this problem, we quantify changes in the toughness, the plateau elastic modulus *G*′, the yield strain *ε*_Y_, and the yield stress *σ*_Y_ that are associated with changes in polymer expression by taking ratios to compare a clinical isolate that has evolved in the lung of a CF patient with its initially-infecting ancestor or, for lab strains, to compare an isogenic mutant with its corresponding wild-type. Each pair of biofilms is grown in parallel, on the same batch of agar plates, and measured on the same day, in immediate succession, to minimize differences in the culture conditions and measurement environment.

### In biofilms grown from clinical isolates, Psl maximizes the energy cost for biofilm disruption

**Fig. 1:**
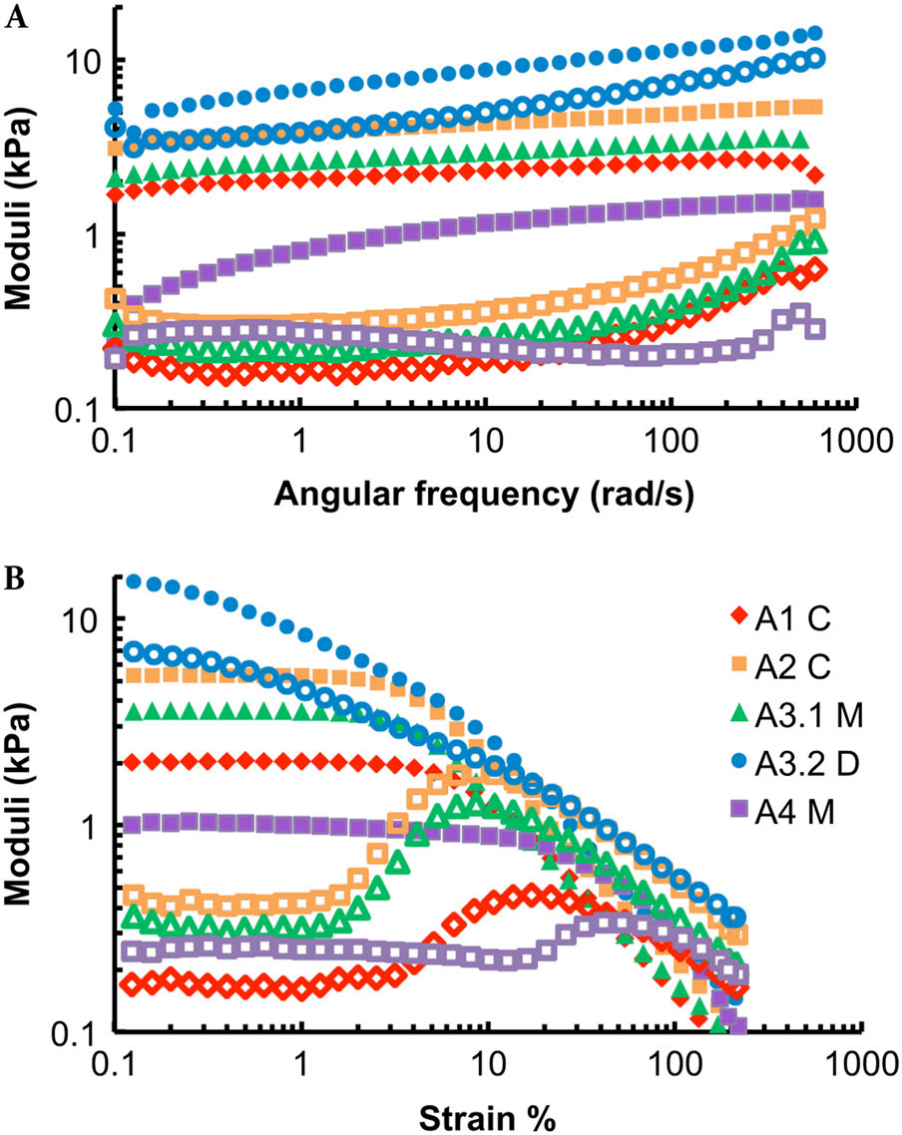
(**a**) Frequency and (**b**) strain sweeps for 1 day’s worth of measurements on biofilms grown from the five infecting strains isolated from Patient A at different points in time. Frequency sweeps were done at 1 % strain and strain sweeps were done at 3.14 radians/s. The elastic moduli (*G*′) are shown with solid symbols and the viscous moduli (*G*′′) are shown with hollow symbols of corresponding shape and color. Strains are listed in order of isolation—see Table S1 for timepoints of isolation. Isolates from later timepoints tend to have higher elastic moduli, except for the two mucoid isolates, A3.1 M and A4 M, which have high alginate production and lower elastic moduli than their immediate ancestors

**Fig. 2:**
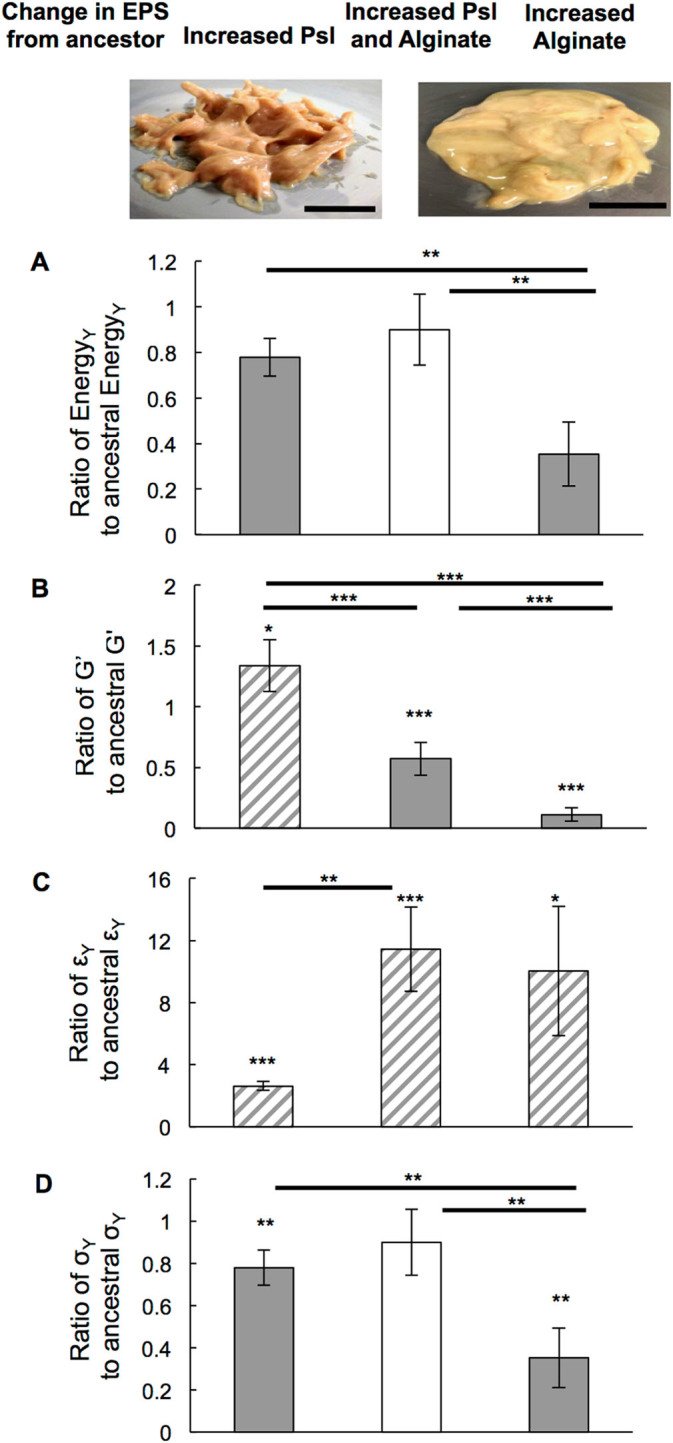
Evolutionary changes in the mechanical properties of biofilms grown from clinical bacterial isolates, distinguished by whether the isolate strain evolved to increase Psl production, increase production of both Psl and alginate, or increase production of alginate, compared with its initially-isolated ancestor. Mechanical changes from the biofilm grown by each strain’s ancestor are measured by taking the ratio of the mechanical property measured for the evolved strain to the corresponding property measured for the ancestor. Thus, a ratio of one indicates no change, a ratio greater than one indicates an increase with evolution, and a ratio less than one indicates a decrease with evolution. Grey highlights diminished mechanics and striped indicates enhanced mechanics. Inset photos show representative images of pooled biofilms that over-express Psl (*left*) and alginate (*right*). The mechanical difference between these two types of biofilms is readily apparent, as increased Psl results in a biofilm that holds the shape of stiff peaks resulting from loading onto the rheometer, but increased alginate results in a biofilm that flows under its own weight to form a smooth surface. (**a**) Increasing production of Psl maintains toughness regardless of whether alginate production increases as well. Increasing production of alginate but not Psl reduces toughness by more than a factor of two. (**b**) Increasing production of Psl, but not alginate, slightly increases the plateau elastic moduli *G*′. Increasing production of alginate but not Psl reduces the plateau elastic moduli G′ by more than 10×. Increasing production of both Psl and alginate results in an intermediate case—biofilms are 1.7× softer than their ancestors but over 6x stiffer than biofilms grown by isolates with increased alginate without increased Psl. (**c**) Increased alginate production increases biofilm yield strains *ε*_Y_ by an order of magnitude, regardless of whether Psl production also increases. Increased production of Psl but not alginate raises yield strain only a few-fold. (**d**) Increased Psl production maintains the yield stress *σ*_Y_, a measure of biofilm strength, at nearly the ancestral value—regardless of whether or not alginate production is also increased. In the absence of increased Psl expression, increased alginate reduces the average yield stress by 2.8×. These data include: seven isolates with increased Psl (but not alginate) production, five isolates with increased production of both Psl and alginate, and four isolates with increased alginate (but not Psl). Each isolate, and its corresponding initially-isolated ancestor, was measured in three independent trials (so *n* = 3) except for strains from patient C, which were measured in two independent trials (*n* = 2). Only two descendent isolates came from patient C—one with increased Psl (but not alginate) and one with both Psl and alginate increased

Energy is the currency of biological processes including phagocytic engulfment, and toughness is a measure of the energy cost, per unit volume, to break or yield a material. To address how evolutionary changes in the production of alginate and Psl impact biofilm toughness, we use oscillatory bulk rheology to measure the shear mechanics of biofilms grown from *P. aeruginosa* strains that were isolated from the sputum of four cystic fibrosis (CF) patients.^16^ (Fig. 1, Figs. S1–S3). These isolates were taken at well-resolved timepoints over ~200–3000 days of infection, and have been genetically and phenotypically characterized to determine the changes arising from in vivo evolution, in particular increases in Psl and/or alginate production (Table S1).^15, 16^ We group isolates by whether production of alginate, Psl, or both has increased from that of the initially-isolated ancestor. For each biofilm studied, both elastic and viscous moduli depend only weakly on the rheometer tool’s frequency of oscillation (Fig. 1a, Figs. S1A–S3A). Strain sweeps at 3.14 radians/s (Fig. 1b, Figs. S1B–S3B) were used to measure the biofilm toughness by integrating stress as a function of strain. We measure changes in toughness by taking the ratio $\frac{{{\rm{Toughness}}\,{\rm{of}}\,{\rm{evolved}}\,{\rm{clinical}}\,{\rm{isolate}}}}{{{\rm{Toughness}}\,{\rm{of}}\,{\rm{initially - isolated}}\,{\rm{ancestor}}}}$ for each pair of biofilms. We find that increasing Psl production is associated with little change in toughness compared with the ancestor, but increasing alginate reduces biofilm toughness to approximately a third of that of the ancestor (Fig. 2a). However, increasing Psl production in combination with alginate production entirely rescues the loss of toughness caused by increased alginate.

The energy cost to cause biofilms to yield is on the order of 10,000 k_B_T/m^3^ for biofilms with increased Psl production, and an order of magnitude less for biofilms with increased alginate production (Fig. S4). For biofilms with increased Psl production, more than 80 % of this energy cost is paid in the form of stored, elastic energy (Fig. S4C), which reflects the fact that these biofilms have plateau elastic moduli *G*′ that are typically ~10 × greater than their plateau viscous moduli *G*′′ (Fig. 1b, Figs. S1B–S3B).

### Psl and alginate make distinct mechanical contributions

Therefore, we dissect the distinct mechanical contributions of each polymer. If Psl production does not increase, we find that increased alginate production decreases the plateau elastic modulus *G*′ by 90 % (Fig. 2b). The yield stress *σ*_Y_ is the product of *G*′ and yield strain *ε*_Y_, which increased alginate increases (Fig. 2c)—but not by enough to prevent the net effect of increased alginate on yield stress being reduction by over 60 % (Fig. 2d). However, if both alginate and Psl production increase, *G*′ decreases by only 40 % (Fig. 2b). For isolates with increased Psl expression, *σ*_Y_ is maintained at roughly the ancestral value, regardless of alginate expression (Fig. 2d). Please note that some of the Psl-overexpressing biofilms begin to yield at the lowest strain measured (e.g., A3.2, D4.2 in Fig. 1, Fig. S3), and as a result our measurements under-estimate the actual stiffening effects of Psl.

Alginate softening biofilms is consistent with others’ work and by our own comparison of ∆*mucA*, an alginate over-producer in the PAO1 background, with the PAO1 wild-type (Fig. S5).^2, 30^ We infer that increasing Psl production can partially counteract softening (decreased *G*′) and entirely counteract weakening (decreased *σ*_Y_) and loss of toughness (decreased Energy_Y_) caused by increased alginate expression.

### Single-bacteria cohesion energies increase with increased Psl production

To probe the role of Psl in biofilm mechanics more directly and at the smallest fundamental unit of the biofilm, we use the cantilever of an AFM to measure force–displacement curves (example shown in Fig. S6) associated with separating matched pairs of isogenic variants of the lab strain PAO1 with well-defined patterns of EPS expression (Table S2). Earlier, we used a similar approach to measure the force of detaching a single bacterium from a surface.^31^ PAO1 in vitro produces Psl and another extracellular polysaccharide, Pel, but does not produce significant amounts of alginate.^32^ We use the ∆*wspF* mutant background of PAO1. The ∆*wspF* mutation results in constitutive high levels of Psl and Pel production due to increased production of the biofilm-promoting, intracellular signal cyclic-di-GMP.

**Fig. 3:**
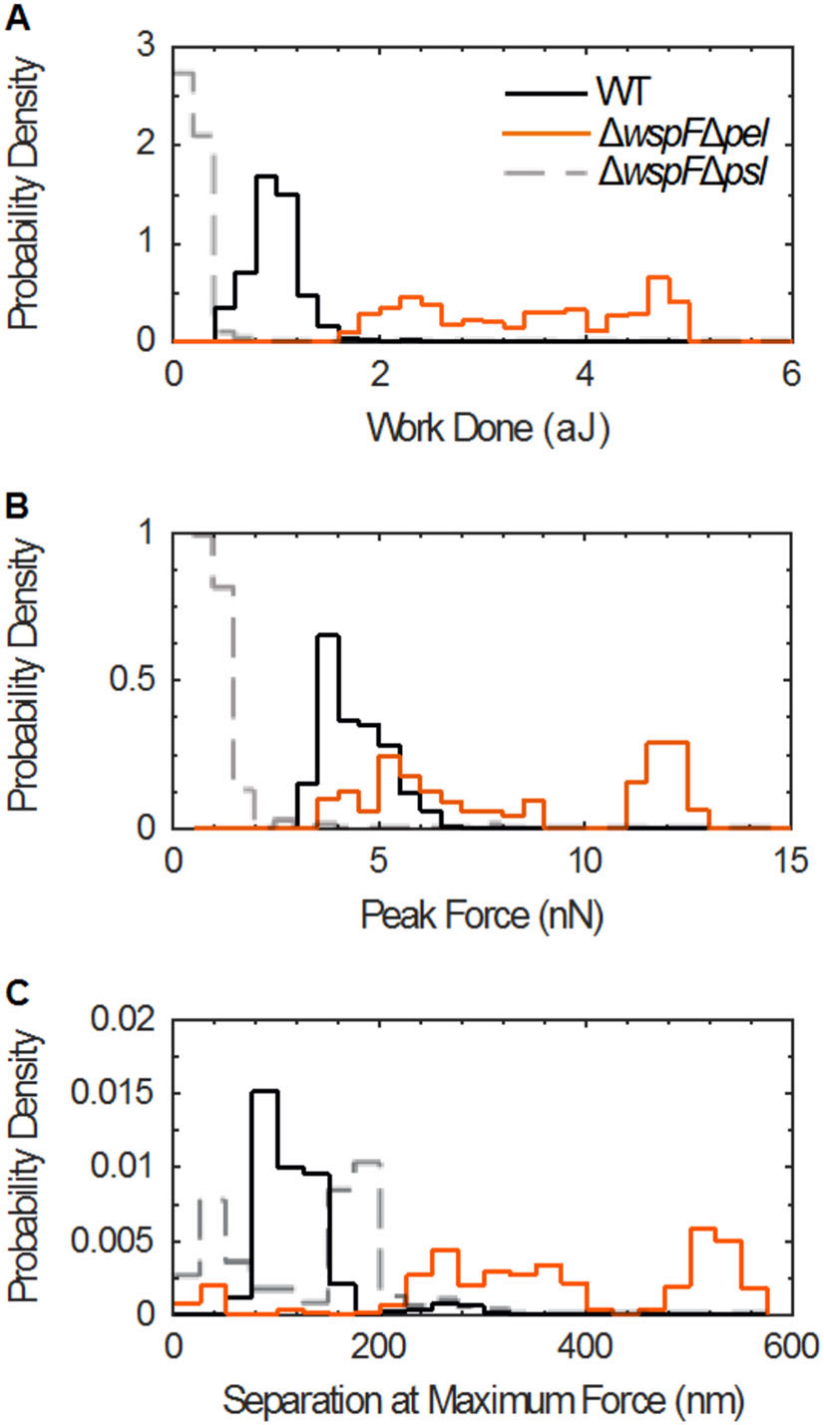
Increased Psl production increases inter-bacterial cohesion. (**a**) Numerical integration of AFM force-displacement curves gives the net mechanical work of detachment. The work for ∆*wspF* ∆*pel* (*solid orange line*) is ~4× greater, on average, than for WT (*solid black line*) and ~10× greater than for ∆*wspF* ∆*psl* (*dashed grey line*). (**b**) The peak force is the maximum force measured for each detachment curve. Peak forces for ∆*wspF* ∆*pel* are greater than for WT and ∆*wspF* ∆*psl*. (**c**) The separation at maximum force is the displacement at which the peak force is found, and is greater for ∆*wspF* ∆*pel* than for WT and ∆*wspF* ∆*psl*. The rate of retraction of the AFM cantilever for the data shown here is 1 μm/s. The trends shown here agree with the trends found when the retraction rate is 10 μm/s (Fig. S9). (*n* = 200–400)

Integrating force over position yields the mechanical work of separation, which measures the energy cost paid to separate two bacteria. The energy cost to separate Psl-overexpressing ∆*wspF* ∆*pel* bacteria is 4–6 times greater than the work to separate wild-type.^2^ (Fig. 3a, Fig. S7A). The increase in energy cost arises secondarily from the larger maximum force applied during detachment (Fig. 3b, Fig. S7B) and primarily from the larger distance over which the inter-bacterial cohesive force is exerted (Fig. 3c, Figs. S7C and D).

### Increased Psl expression stiffens and toughens biofilms grown from the lab strain PAO1

**Fig. 4:**
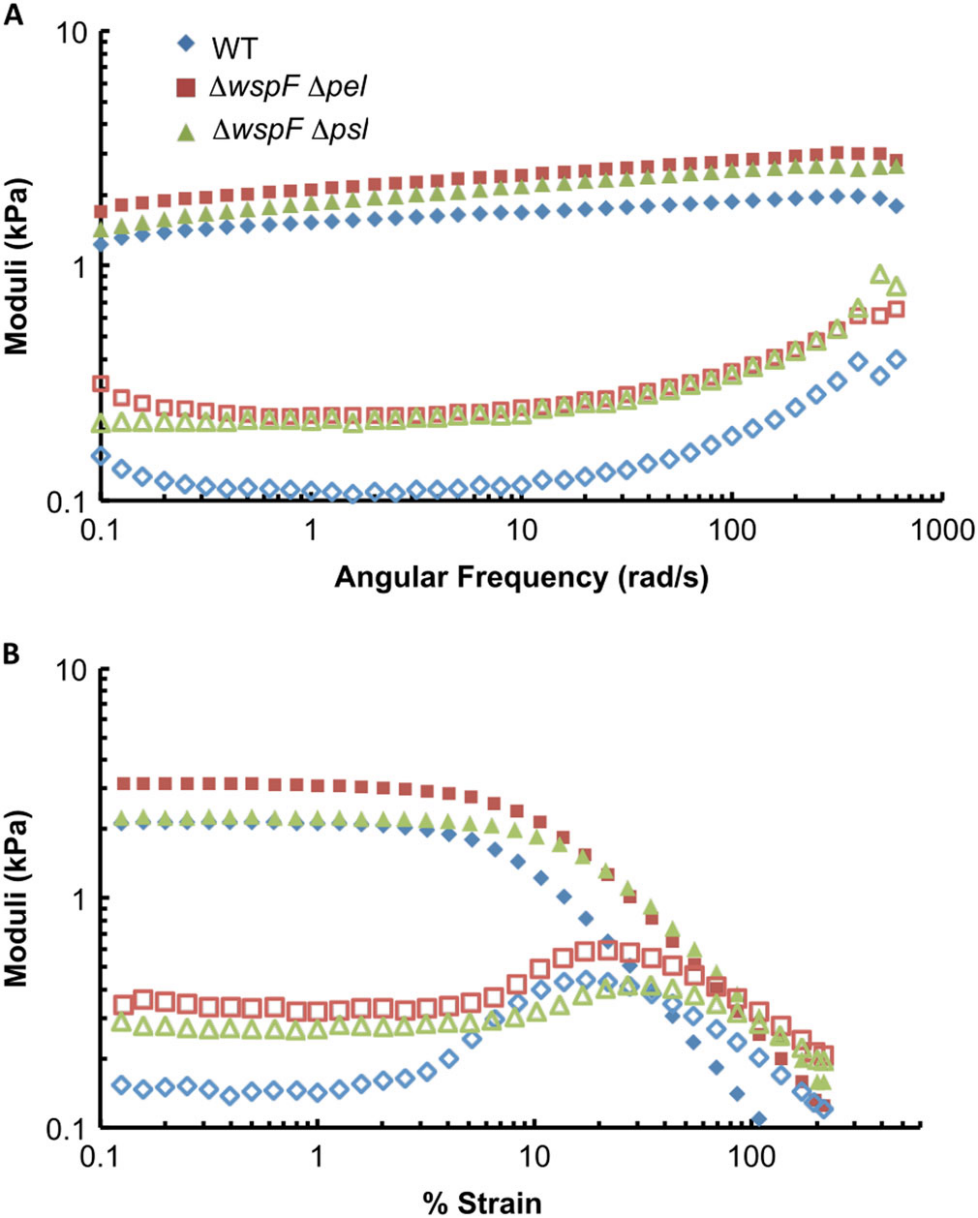
Representative (**a**) frequency and (**b**) strain sweeps from 1 day‘s worth of measurements on biofilms grown from lab strains of bacteria. Frequency sweeps are at 1 % strain and strain sweeps are at 3.14 radians/s. Elastic moduli (*G*′) are shown with solid symbols and viscous moduli (*G*′′) are shown with hollow symbols of the corresponding color and shape. Compared with the wild-type bacteria, ∆*wspF* ∆*pel* produces higher amounts of Psl (and does not produce Pel) and ∆*wspF* ∆*psl* produces higher amounts of Pel (and does not produce Psl). Increased Psl production results in an increase in the plateau elastic modulus. Increased Pel production results in an increase in the yield strain

**Fig. 5:**
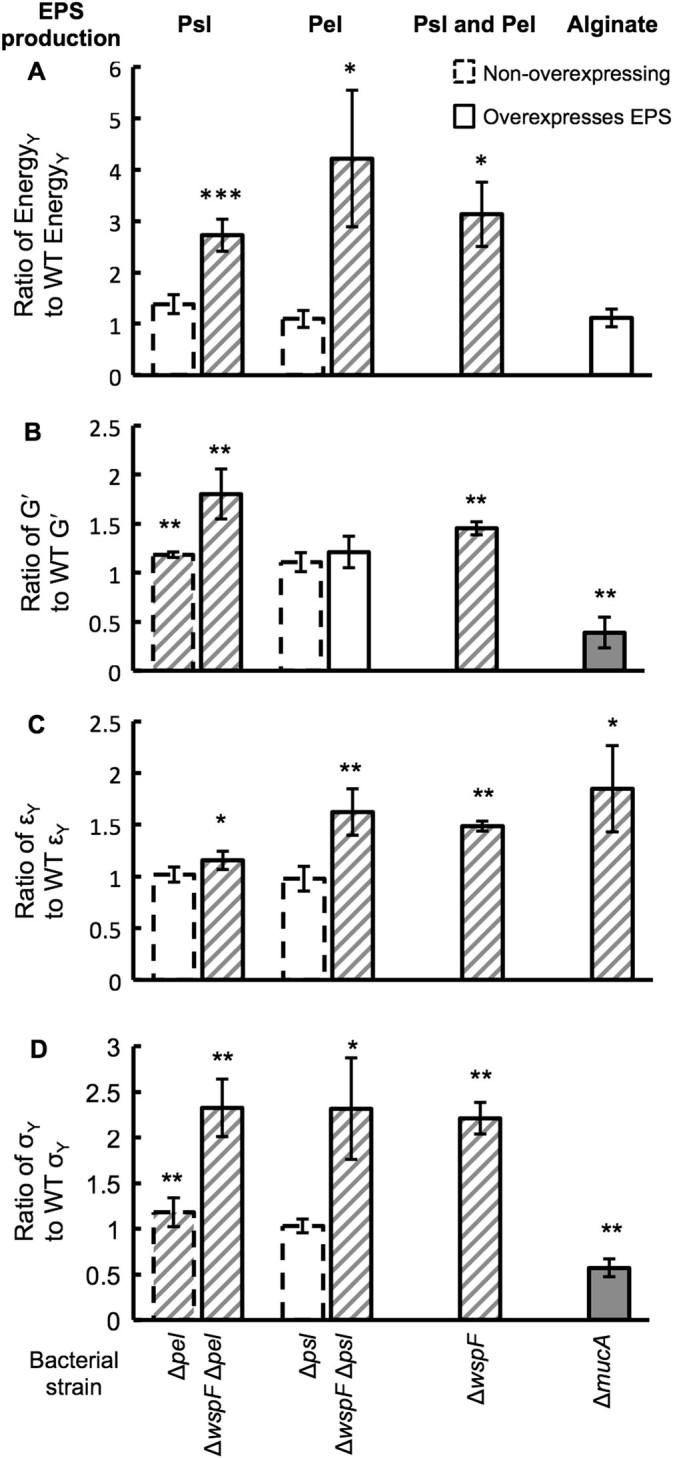
Changes in PAO1 biofilm mechanics associated with changes in EPS expression are measured by taking the ratio of the value for a mutant biofilm to the value for the WT biofilm. Striped highlights enhanced mechanics and grey highlights diminished mechanics. Error bars are standard error of the mean. (**a**) Increasing the production of Psl or Pel, as seen in ∆*wspF* ∆*psl* (*n* = 4)*,* ∆*wspF* ∆*pel* (*n* = 4)*,* and ∆*wspF* (*n* = 2), increases biofilm toughness. Increasing the production of alginate (Δ*mucA*) has no net effect on biofilm toughness (*n* = 3). Please note that our measurement of toughness as the energy required to cause the biofilm to yield includes both elastic and viscous contributions, while the properties in panels (**b**–**d**) are all elastic properties. (**b**) Increased production of Psl (∆*pel* (*n* = 3), ∆*wspF* ∆*pel* (*n* = 4), and ∆*wspF* (*n* = 2)) causes biofilms to have higher plateau elastic moduli *G*' than does the WT biofilm. Increased production of alginate reduces plateau value of *G*′. Increased production of Pel without a concomitant increase in Psl (∆*psl* (*n* = 3), ∆*wspF* ∆*psl* (*n* = 4)) does not impact *G*′. (**c**) Increased production of Pel or alginate increases the yield strain *ε*_Y_. Over-expression of Psl results in, at most, a minor increase in yield strain. (**d**) Over-expression of Psl and/or Pel increases biofilm yield stress *σ*_Y_. Over-expression of alginate reduces yield stress

To confirm the connection between single-bacteria cohesion mechanics to bulk biofilm rheology, we performed rheological tests on biofilms grown using the isogenic lab strains used for AFM measurements (Fig. 4) and on their isogenic single-gene-knockout counterparts (Fig. S8). We quantify changes in mechanical properties associated with changes in EPS production by taking ratios comparing an isogenic variant with PAO1 WT (Fig. 5). We find that increased production of Psl consistently increases the energy cost to break the biofilm—i.e., the biofilm toughness is increased—with the lowest *p*-value value of any of our tests on any lab strain (Fig. 5a). The energy cost to break these lab-strain biofilms is approximately 1000 k_B_T/m^3^, less than for biofilms grown from clinical isolates (Fig. S9A).This largely reflects our finding that some clinical-strain biofilms have higher viscous moduli than do lab-strain biofilms (Figs. 1 and 4, Figs. S1–S3, S8). The elastic energy cost paid to cause lab-strain biofilms to yield is typically 10× greater than the viscous energy cost (Fig. S9B).

### Increased Psl or Pel expression strengthens PAO1 biofilms

Therefore, we examine the contributions of each polymer to specific elastic mechanical properties of biofilms. We find that ∆*wspF* ∆*pel* biofilms, with high amounts of Psl, have plateau *G*′ 80 % greater than that of biofilms grown from WT (Fig. 5b)*.* Furthermore, biofilms of ∆*pel* have plateau *G*′ 20 % greater than that of the WT biofilm. It is likely that the higher plateau *G*′ for ∆*pel* reflects an increase in Psl production when Pel production is eliminated.^33^ In contrast, we find that over-expression of Pel increases the biofilm’s yield strain *ε*_Y_ by 60 % (Fig. 5c), while leaving the plateau value of *G*′ unchanged. These changes are unlike those found for increased alginate, which increases yield strain while decreasing elastic modulus. Increased Psl production has some impact on yield strain, but not as much as increased Pel. Biofilms grown from ∆*wspF* bacteria, which over-express both Psl and Pel, have *G*′ that is 50 % greater than that of WT biofilms and a yield strain that is 44 % greater than that of WT biofilms.

For WT PAO1 biofilms, yield stress is typically ≈15 kPa. We find that the yield stress is 130 % greater for both ∆*wspF* ∆*pel* and ∆*wspF* ∆*psl* (Fig. 5d)*.* ∆*wspF* biofilms have yield stress that is, on average, 50 % greater than that of biofilms grown from WT. From this we conclude that increased expression of either Psl or Pel can increase the material strength of *P. aeruginosa* biofilms, although Psl does so by stiffening the biofilm and Pel does so by making the biofilm more ductile. Over-expression of both (by *∆wspF*) results in comparable strengthening to that for either one alone.

### Psl likely stiffens biofilms because it is cross-linked by the protein CdrA

It is striking that, of the three EPS materials examined, Psl is the only one that acts to stiffen the biofilm when its production increases. Furthermore, examining mature biofilms (grown in flow cells) under confocal and phase contrast microscopes shows a low volume fraction of discrete bacteria bound in a large, continuous, primarily-polymer matrix that is well over 50 % of the volume of the biofilm. Therefore, we expect that changes in biofilm mechanics are most likely to represent changes in the polymer matrix of the biofilm, not changes in the interactions between bacteria and the matrix.

**Fig. 6:**
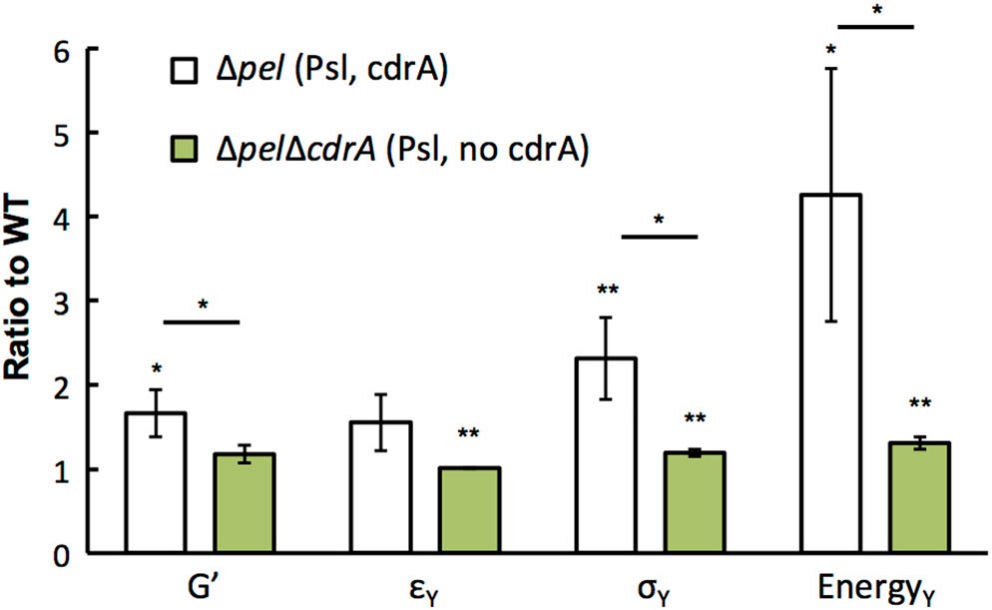
Production of the protein CdrA is needed for the stiffening (increased *G*′), strengthening (increased *σ*_Y_), and toughening (increased yield energy Energy_Y_) effects of Psl. For Δ*pel* strains that produce Psl but not Pel, biofilm mechanics are enhanced compared to the WT only if CdrA is also produced. (*n* = 3)

It is well-known that increasing the concentration of polymer (*c*) in a gel will increase the gel’s stiffness, *G*′∝ *c* ^*A*^, where *A* is a scaling factor that is 2.25 for entangled polymer in good solvent.^34^ Stiffening by increasing polymer concentration is a physical effect that does not depend on polymer chemistry. However, increasing Pel and alginate production does not stiffen biofilms. Moreover, our biofilms are grown in contact with a large water reservoir (the agar gel), which should act to minimize differences in polymer concentration. Thus, we infer that specific chemical characteristics of the EPS types govern their different effects on biofilm mechanics. Psl is a neutral, branched pentasaccharide made of D-glucose, D-mannose, and L-rhamnose.^35^ Psl contains several mannose groups, including a mannose side-chain, to which the protein CdrA, which has been suggested by other researchers as a possible crosslinker, binds.^21, 33^ CdrA is co-regulated with Pel and Psl through c-di-GMP induction, and is therefore over-produced in all the *∆wspF* backgrounds. To examine the role of CdrA in contributing to stiffening by Psl, we measure the shear mechanics of biofilms grown from ∆*pel* ∆*cdrA*, which does not produce CdrA, and compare with ∆*pel* biofilms. When the capacity to make CdrA is lost, the stiffening effect found for ∆*pel* is lost and the elastic modulus and toughness return to approximately those of the WT (Fig. 6). From this, we conclude that CdrA likely crosslinks Psl and that this is the cause of Psl-induced stiffening. Furthermore, our AFM measurements imply that stiffening by Psl should result from a process that can occur over the 1 s during which the two bacteria are in contact before we begin separation. Chemical processes, such as protein binding, are faster than physical process such as polymer entanglement. Indeed, if reptation of entangled polymers were important for our systems over the timescales of measurement, we would expect to see a crossover in viscous and elastic moduli at the inverse of the reptation time; instead, no such crossover is seen for frequencies from 0.1 to 1000 Hz (Fig. 4a, Fig. S8A).

## Discussion

### Summary of results

Increasing the expression of the extracellular polysaccharide Psl increases the stiffness and the mechanical strength and toughness of *P. aeruginosa* biofilms. This must arise from molecular specifics of Psl because increasing the expression of other extracellular polysaccharides, namely Pel and alginate, or increasing Psl without the presence of the Psl-binding protein CdrA, have different effects on biofilm mechanics. Increasing the expression of Pel also strengthens the biofilm, but by increasing yield strain rather than increasing stiffness. In clinical isolates, increasing alginate expression softens and weakens the biofilm, but this can be counteracted by also increasing Psl expression, which synergizes with increased alginate expression to increase the energy cost to break the biofilm. Thus we have shown that, in CF lungs, biofilms have a tendency to evolve in such a way that mechanical toughness and stiffness are maintained, despite a parallel tendency to evolve increased alginate production, which weakens and softens biofilms.

### Potential impact on clearance by the immune system

Neutrophils are phagocytic immune cells that densely surround biofilm infections without actually entering the biofilm.^36, 37^ It has been recently pointed out that, because ~10 μm neutrophils are an order of magnitude smaller than ~100 μm biofilm infections, the only way that neutrophils will be able to engulf biofilm bacteria will be if they can break off a small piece of the biofilm.^38^ During phagocytosis, neutrophils exert estimated attractive stresses of ~1 kPa.^39^ The elastic moduli *G*′ that we measure are also on the order of ~1 kPa (Fig. 1, Figs. S1–S3). Since the elastic moduli we measure are comparable to the stresses exerted by neutrophils during phagocytosis, it is plausible that the tenfold difference in *G*′ and the over-twofold difference in yield stress that we measure for biofilms with different patterns of matrix production could impact their susceptibility to phagocytosis and that increasing Psl production could help to protect against phagocytosis through its impact on *G*′ and yield stress. In addition to overall susceptibility, biofilm mechanics are likely to impact the timescale of phagocytosis. Alterations in timescale matter because bacterial biofilms can express virulence factors that kill neutrophils and other immune cells—so that delays that give more time for these biochemical factors to act should be protective for the biofilm, even if the mechanical changes per se do not altogether prevent phagocytosis. Thus, biofilms may act as a fortress to resist mechanical attack.

### Mapping in vitro properties onto in vivo properties

It is widely thought that the mucus in CF airways is more concentrated, with less water, compared with the mucus in normal, healthy airways (for examples of an extensive literature containing a great deal of controversy, see Garland et al. 2013 PNAS 110:15973–15978 and Mall et al. 2004 Nature Medicine 10:487–493^40, 41^). Although *P. aeruginosa* biofilms are not typically found in healthy lungs, it is possible that if they were, they would be more hydrated than *P. aeruginosa* biofilms of the same strain in CF lungs. The degree of such difference in hydration, if any, is not known.

The lungs consist of the respiratory zone, which does not have submucosal glands, and the conductive zone, where mucus is produced and the mucociliary escalator provides a defense against pathogens by removing them.^42^ This removal is hindered in CF because of the high viscosity of concentrated mucus. In terms of CF lung infections, *P. aeruginosa* biofilms are rarely found in the respiratory zone of mature patients (like the ones from which our clinical isolates were drawn), likely owing to the intensive antibiotic treatment commonly used for this disease.^42^ Sputum, from which all the clinical isolates we study were taken, is coughed up from the conductive zone of the lungs, so that any biofilms growing in it are embedded in mucus. Because the mucus volume is much greater than the volume of the ~100 μm biofilm spheroids found in CF lungs,^36^ the mucus acts as a fluid reservoir for the biofilms just as does the nutrient agar gel in our petri dishes on which we grow biofilms. Therefore, to zeroth order, we expect the degree of hydration for all biofilms growing within the conductive zone to be roughly the same, *modulo* any inter-patient differences in mucus composition and any intra-patient changes in mucus composition over time. We therefore expect the shifts in mechanics we measure in vitro to result from different biofilm matrix compositions to correspond to similar trends of biofilm mechanics in vivo.

### Related and future work—microrheology

Our finding that Psl stiffens biofilms and Pel makes biofilms more ductile are reasonably congruent with a recent microrheological study that examined the effects of Pel and Psl on the creep compliance of biofilms grown in flow cells using an alginate-overexpessing background.^43^ Creep compliance describes the deformation of a material under a constant load and it includes both elastic (solid-like) and viscous (fluid-like) interactions. In contrast, our use of oscillatory bulk rheology allows us to distinguish the elastic modulus *G*' and the viscous modulus *G′′*. Passive microrheology is well-adapted to studies of local heterogeneities, which our bulk measurements cannot probe, but passive thermal motions are insufficient to cause biofilms to yield. Therefore, passive microrheology cannot obtain information about yielding and toughness. Thus, our measurements also allow us to compare the elastic modulus and yield stress of biofilms grown from clinical strains with the stresses other researchers have estimated that phagocytosing neutrophils exert, to show that the mechanical changes we measure and associate with different evolutionary changes in polysaccharide production are likely to have a significant impact on the biofilm infections’ susceptibility to phagocytosis. This is the first hint of evolution to adapt biofilm mechanics under pressure from the immune system.

### Related and future work—Pel electrostatic binding to DNA

Unlike our bulk measurements of Pel-overexpressing biofilms, our AFM studies of cell–cell cohesion show that Pel over-expression actually decreases the distance over which inter-bacterial cohesion forces are exerted (Fig. 3c, Figs. S7C and D) and the work of detachment (Fig. 3a, Fig. S7A), compared with the WT. Thus, unlike Psl, the effects of Pel on biofilm mechanics are inconsistent with its effects at the single-cell level. This implies that the increased ductility and the resulting increased yield stress of Pel-overexpressing biofilms is an emergent property of the biofilm state. Recent work has shown that Pel is cationic and binds extracellular DNA.^44^ Extracellular DNA is a significant component of the polymeric matrix in biofilms,^45^ but not for the planktonic bacteria in our AFM studies.Therefore, we speculate that the increased biofilm yield strain, and resulting greater yield stress, associated with increased Pel production arise from Pel binding to extracellular DNA. For example, in so-called “double network” gels made of two kinds of interacting polymers, the yield strain can be greater than that of a gel made of either polymer alone, as a result of mobile inter-polymer junctions and of one polymer acting as “hidden length” within the network.^46–53^

### Related and future work—heterogeneity

Other researchers have shown that the spatial distribution of polysaccharide types in biofilms is heterogeneous.^44, 54, 55^ Integrating this with our results implies that biofilm mechanics are likely to be heterogeneous, as seen for *E. coli*.^56^ Thus, the localized effects of specific polymers may be much greater than our bulk rheology measurements are able to characterize. Future work, using microrheology^43, 56^ in combination with staining for specific EPS materials, could address this. Furthermore, although our studies have focused on monoclonal biofilms, real biofilm infections are often poly-clonal and poly-species, which increases the number of ways in which biofilms could be heterogeneous and tuned for optimum fitness.

## Methods

### Bacterial strains and growth conditions

The strains of *P. aeruginosa* used in this study are described in Table S1 (for clinical isolates^15, 16, 57^) and Table S2 (for lab strains^17, 21, 58–61^). Each lab strain constitutively expresses green fluorescent protein (GFP). GFP expression was intended as future-proofing so that we could later examine the relationship between single-cell behavior imaged using fluorescence microscopy and the bulk biofilm rheology that we measure here.

We grew all bacterial cultures in Luria broth (LB). For rheology, strains were grown overnight in 4 mL LB, shaking at 37 °C. Then we spread the overnight culture onto agar plates at 250 μL/plate. The biofilm grew overnight on the agar plates.

### Background—rheology

Mechanically, biofilms are viscoelastic materials,^24^ with both solid-like (elastic) and fluid-like (viscous) properties. We measure the bulk mechanics of biofilms by applying an oscillatory shear strain, *ε* = *ε*_0_sin(*ωt*) (where *ω* is the angular frequency of oscillation and *t* is time) and measuring the resulting shear stress using a rheometer.^27^ Shear strain is the lateral deformation of a material divided by its thickness in the non-deformed direction (Figs. S10A and B).

Stress is force per unit area and is the product of the strain and the elastic modulus. When a strain is applied, the material’s stress response,

1 $$\sigma {\rm{ = }}{{\rm{\sigma }}_{\rm{0}}}{\rm{sin}}\left( {\omega t{\rm{ + }}\delta } \right){\rm{ = }}{{\rm{\varepsilon }}_{\rm{0}}}{\rm{[}}G^\prime {\rm{sin}}\left( {\omega t} \right){\rm{ + }}G^{\prime\prime} {\rm{cos}}\left( {\omega t} \right){\rm{],}}$$

gives the elastic modulus *G*′ and the viscous modulus *G*′′. The viscous modulus characterizes the material’s fluid-like resistance to flow. The elastic modulus characterizes the material’s solid-like resistance to deformation in the elastic regime where the stress–strain relationship is linear and reversible (Figs. S10C and D). When a material is deformed past the elastic regime, the material is irreversibly deformed and begins to mechanically fail. The yield strain is the strain at which material failure begins, and the yield stress is the stress at which material failure begins (Figs. S10C and D).

### Rheology measurements

We used a stress-controlled TA Instruments AR-G2 Magnetic Bearing Rheometer for bulk rheology measurements. We used a parallel-plate tool geometry, with a roughened surface to prevent slippage. The biofilm from multiple agar plates (grown from the same strain) was scraped directly onto the rheometer using a metal spatula. We typically used 10–15 plates’ worth of biofilm per measurement. Biofilm was loaded into the rheometer tool as quickly as possible, with two people working together, to minimize evaporation. Then, the geometry of the rheometer was lowered to a 500 μm gap height, and any excess biofilm was trimmed, if needed, so that sample material did not extend beyond the edge of the tool. Filling the tool with this gap required approximately 0.6 mL of biofilm. Since the 500 μm gap height is smaller than recommended for this rheometer, we tested the output of the rheometer at this gap height in the regime of our measurements with a calibration oil to verify that this gap is acceptable. To prevent drying of the biofilm during measurement, a cylindrical solvent trap was made from polycarbonate, lined with moist cotton balls, and placed around the geometry and base of the rheometer. An image of the solvent trap and time-sweeps contrasting the effects of drying, in the absence of the solvent trap, with no drying, when the solvent trap is used, are shown in Fig. S11.

Oscillatory frequency sweeps from 0.1 to 600 rad/s at 1 % strain and strain sweeps from 0.1 to 200 % at 3.14 rad/s were performed on each sample on each day of measurement. The plateau modulus G′ was taken as the elastic modulus at 1 % strain for biofilms grown from lab strains of bacteria, and at 0.5 % strain for biofilms grown from clinical isolates. These strain values are below the yield stress for the biofilms measured—a lower strain was used for biofilms grown from clinical isolates because many of these yielded before 1 % strain.

Representative frequency and strain sweeps are shown for 1 day’s worth of measurements on clinical isolates (from one patient) in Fig. 1 and Figs. S1–S3, and for WT and EPS over-expressing lab strains in Fig. 4 and Figs. S5 and S8.

### Determination of yield strain and stress

The yield strain of each biofilm was determined from strain–sweep data. The plateau region on the left part of each strain–sweep graph, where stress and strain are linearly related, was fit to a linear equation. The region on the right part of each strain–sweep graph, after the biofilm has “broken” and the modulus quickly decreases with increasing strain, was fit to a power law. The strain at which these equations intersect was taken to be the yield strain (Fig. S10D). The corresponding yield stress was then determined from the raw rheology data.

### Statistical significance

*P*-values are calculated using a Student one-tailed *t*-test. Each *p*-value associated with a single bar tests the null hypothesis that the ratio should be unity. Each *p*-value that compares two bars tests the null hypothesis that the compared values are the same. *P*-values are indicated on the figures as follows: *p < 0.1,**p < 0.05,***p < 0.005. Error bars are standard error of the mean. The sample size and number of repetitions was chosen to give *p*-values of 0.05 or less for our central conclusions (Figs. 2, 5, and 6). In figures, error bars are standard error of the mean.

### AFM force measurements

We used a BioScope Catalyst BioAFM (Bruker) for AFM measurements. This instrument combines both fluorescence and atomic force microscopy. We used triangular silicon nitride cantilevers (Bruker, MSNL-10, spring constant 0.07 N/m) for all measurements. Thermal tuning was used to confirm the cantilever spring constant. The cantilevers were loaded into a fluid probe holder.

To prepare the probe for attachment of bacteria, we dipped the probe into a droplet of 0.1 % w/v poly-L-lysine solution for approximately 30 min. The probe was removed from the droplet and the excess solution was wicked from the cantilever using a Kimwipe. Poly-L-lysine is a polycation that electrostatically attaches bacteria to the probe. We allowed the probe to dry for 10 min and then dipped it into a droplet of bacterial suspension. The probe was in the bacterial suspension for ~30 min and was then gently rinsed with deionized water. We verified the presence of bacteria on the cantilever using a Plan Apo 60× oil objective (Nikon) with bright-field microscopy.

While the cantilever was being prepared, a glass slide was prepared similarly. We placed a droplet of 0.1 % w/v poly-L-lysine onto the glass slide for 30 min; we wicked away excess solution with a Kimwipe; the slide was allowed to dry for 10 min. A droplet of bacterial suspension was then placed onto the same location, left there for 30 min, and then rinsed. We then placed a fresh droplet of de-ionized water on the fixed bacteria.

We used the AFM probe, with attached bacteria, to image the surface in contact mode. While the presence of bacteria on the probe decreases image quality, the location of bacteria on the surface can still be discerned from the resulting image. Once bacteria were located on the surface, the probe was positioned over a single bacterium on the surface. We lowered the cantilever tip onto a bacterium, held the tip on the bacterium for 1 s, and then retracted the probe. This cycle was repeated 200–400 times over a vertical distance of 4 μm at speeds of both 10 μm/s (Fig. S9) and 1 μm/s (Fig. 3). We used the deflection of the cantilever upon retraction to measure the adhesion force between the bacterium on the surface and the bacterium on the cantilever. An annotated representative force–displacement curve is shown in Fig. S6.

### Analysis of force–displacement curves

We used MATLAB to analyze the data from the retraction curves obtained using the AFM. Frequently, bacteria were removed from the surface during over the course of hundreds of approach-contact-retraction cycles, and sometimes the AFM tip would break over the course of cycles. To distinguish true inter-bacterial force-separation curves, we discarded all the curves which had an interaction region less than 150 nm (that of the “control curve” taken with a tip attached to bacteria, and a surface free of bacteria). We also discarded all the plots which have a consistent slope till the minima in the interaction region; if we are measuring a polymer interaction rather than the sensitivity of the cantilever, we would expect the slope to change once the tip begins to pull off the surface.

Using the measured spring constant of the cantilever, the slope extracted from the contact region is used to calculate the force exerted on the cantilever during retraction. The work done during retraction was calculated by numerical integration in Matlab. The quantities measured are roughly the same for a cantilever tip retraction speed of 1 μm/s (Fig. 3) or 10 μm/s (Fig. S9).

## Electronic supplementary material

Supplementary Information

Figure S1

Figure S2

Figure S3

Figure S4

Figure S5

Figure S6

Figure S7

Figure S8

Figure S9

Figure S10

Figure S11
