# Supplementary material for: Evolutionary adaptations of biofilms infecting cystic fibrosis lungs promote mechanical toughness by adjusting polysaccharide production
Source: NPJ Biofilms Microbiomes. 2017 Jan 23;3:1. doi: 10.1038/s41522-016-0007-9 (PMC5445605; doi:10.1038/s41522-016-0007-9)
Supplement: Supplementary file 1 — Supplementary Information [file 41522_2016_7_MOESM1_ESM.docx]

**Supplementary Tables**

**Table S1: Clinical isolates used in this study** Chronological clinical isolates from four cystic fibrosis patients with chronic *P. aeruginosa* infections (Patients A-C [[1](#_ENREF_1)]; Patient D [[2](#_ENREF_2)]) were used in this study. To bin data for the analysis shown in Figure 3, each strain used except for the initially-isolated ancestor strain was classified as having increased Psl, increased alginate, or both, compared with the ancestor. Increased alginate was determined by visual identification of mucoid colonies. Determination of increased Psl was taken from [[3](#_ENREF_3)]. Isolates that had neither increased Psl nor increased alginate were not used because the purpose of this study was to examine the effect of changes in EPS production on biofilm mechanics.

| Label | Sample ID | Days *in vivo* | Colony morphology | EPS classification |
| --- | --- | --- | --- | --- |
| A1 | C2773C | 0 | Classic | Ancestor |
| A2 | C3470C | 570 | Classic | Not used |
| A3.1 | C3639M | 703 | Mucoid | Increased Psl and Alginate |
| A3.2 | C3640D | 703 | Small | Increased Psl |
| A4 | C4278M | 1194 | Mucoid | Increased Psl and Alginate |
| B1 | C1913C | 0 | Classic | Ancestor |
| B2.1 | C4218C | 1669 | Classic | Increased Psl |
| B2.2 | C4219D | 1669 | Small | Increased Psl |
| B2.3 | C4220M | 1669 | Mucoid | Increased Psl and Alginate |
| B3.1 | C5912M | 2790 | Mucoid | Increased Alginate |
| B3.2 | C5913C | 2790 | Classic | Not used |
| B3.3 | C5914M | 2790 | Mucoid | Increased Alginate |
| Cb1 | C2159M | 0 | Mucoid | Ancestor |
| Cb2 | C3488D | 1025 | Small | Increased Psl |
| Cb3 | C5623M | 2545 | Mucoid | Increased Psl and Alginate |
| D1 | C3881C | 0 | Classic | Ancestor |
| D2.1 | C4197D | 252 | Small | Not used |
| D2.2 | C4198C | 252 | Classic | Increased Psl |
| D3.1 | C6926M | 1988 | Mucoid | Increased Psl and Alginate |
| D3.2 | C6927C | 1988 | Classic | Increased Psl |
| D4.1 | C7514E | 2394 | Entire | Not used |
| D4.2 | C7515D | 2394 | Small | Increased Psl |

**Table S2: Lab strains used in this study.**

| Strain | Description | Ref. |
| --- | --- | --- |
| PAO1 GFP | Wild Type (WT) makes Psl and some Pel, no alginate | [[4](#_ENREF_4)] |
| Δ*pel* GFP | Makes Psl, no Pel, no alginate | [[5](#_ENREF_5), [6](#_ENREF_6)] |
| Δ*psl* GFP | Makes Pel, no Psl, no alginate | [[7](#_ENREF_7)] |
| Δ*mucA* GFP | Over-expresses alginate | [[8](#_ENREF_8)] |
| Δ*wspF* Δ*pel* GFP | Over-expresses Psl, no Pel, no alginate | [[9](#_ENREF_9)] |
| Δ*wspF* Δ*psl* GFP | Over-expresses Pel, no Psl, no alginate | This paper (for GFP addition) |

**Supplementary Figure Legends**

**Figure S1**. Representative (A) frequency sweeps and (B) strain sweeps at 3.14 radians/s for biofilms regrown from clinical isolates taken from Patient B. Elastic moduli are shown by filled symbols and viscous moduli are shown by the corresponding hollow symbols. For most of these clinical isolates, the elastic modulus G′ of is greater than its viscous modulus G′′. However, the ratio G′′/G′, and therefore tan(δ), where δ is the phase constant in Equation 1, is much more varied for clinical isolates than for PAO1 lineages, and for B3.1 M and B3.3 M at some frequencies and strains, G′′/G′ is approximately unity and our approximation that biofilms are predominantly solid-like breaks down.

The legend lists strains in order of isolation – see Table S1 for timepoints of isolation. Isolates from later timepoints in the infection’s history that have converted to the mucoid phenotype (designated by M) tend to have lower elastic moduli than do isolates from earlier timepoints. Exceptions are found for isolates that also have increased Psl expression.

Data points for the elastic response of bacterial strain B1 C are hidden under data points for bacterial strain B2.1 C, and data points for the elastic response of bacterial strain B2.2 C are hidden under data points for bacterial strain B3.2 C. Data points for the viscous response of bacterial strain B2.2 C are largely hidden under data points for bacterial strains B2.3 M and B3.3 M.

**Figure S2**. Representative (A) frequency sweeps and (B) strain sweeps at 3.14 radians/s for biofilms regrown from clinical isolates taken from Patient C. Elastic moduli are shown by filled symbols and viscous moduli are shown by the corresponding hollow symbols. Data points for the elastic response of bacterial strain Cb1 M are largely hidden under data points for bacterial strain Cb2 D. For bacterial strain Cb3 M, the ratio G′′/G′ is of order unity, indicating that our approximation of biofilms as elastic solids breaks down for this clinical isolate.

**Figure S3**. Representative (A) frequency sweeps and (B) strain sweeps at 3.14 radians/s for biofilms regrown from clinical isolates taken from Patient D. Elastic moduli are shown by filled symbols and viscous moduli are shown by the corresponding hollow symbols. Data points for the elastic response of bacterial strain D1 are largely hidden under data points for bacterial strain D2.2.

**Figure S4**. Evolutionary changes in the toughness of biofilms grown from clinical bacterial isolates, broken down by increased Psl and alginate production. Isolates from timepoints after the first are categorized as having increased Psl expression, increased expression of both Psl and alginate, or increased alginate expression. Grey highlights diminished mechanics, and orange-and-grey striped indicates diminished mechanics that are greater than for increased alginate alone. (A) Increasing production of both Psl and alginate increases the energy cost to break the biofilm more than increasing either polysaccharide alone. (B) Biofilms grown from strains that have increased production of Psl have greater breaking energy density than biofilms that increase production of alginate only. (C) The contribution to breaking energy that comes from elastic (stored) energy contributions is typically 3-6× greater than the contribution that comes from viscous (dissipated) energy contributions.

**Figure S5.** Representative strain sweeps at 3.14 radians/s for WT and Δ*mucA* biofilms. Elastic moduli are shown by filled symbols and viscous moduli are shown by corresponding hollow symbols. Notably, for Δ*mucA,* the ratio G′/G′′, while still greater than unity, is much less than for WT or for the non-alginate lab strains measured in Figures 3 and S10. This suggests that alginate expression is likely to be a factor in the variability of the ratio of elastic modulus to viscous modulus for clinical isolates (Figure 1, Figures S1-S3).

**Figure S6**. A representative force-displacement curve associated with detaching two Psl over-producing bacteria. At (A), the AFM cantilever tip begins to lift from the surface. At (B), the greatest force magnitude is measured; the distance between (B) and (A) is the separation at maximum force. At (C), the bacterium detaches completely. The distance between (C) and (A) is the interaction range.

**Figure S7**. Histograms of quantities measured by separating two matched bacteria with an AFM. The rate of retraction of the AFM cantilever is 10 μm/s. As with the data in Figure 3, taken with a retraction speed of 1 μm/s, overall, Δ*wspF* Δ*pel* show stronger mechanical interaction and greater variability than WT and Δ*wspF* Δ*psl*. (A) Numerical integration of force-displacement curves gives the net mechanical work of detachment. (B) The peak force is the maximum force measured for each detachment curve. (C) The separation at maximum force is the displacement at which the peak force is found. (D) The range measures the size of the displacement undergone before the force returns to its baseline, zero value. This measures the lengthscale over which two bacteria can be separated but still mechanically interacting.

**Figure S8.** Representative frequency and strain sweeps measuring elastic and viscous moduli for WT, ∆*pel*, and ∆*psl* biofilms. Elastic moduli are shown by filled symbols and viscous moduli are shown by the corresponding hollow symbols. (A) Frequency sweep run at 1% strain. (B) Strain sweep run at 3.14 radians/s.

**Figure S9**. (A) Energy density required to cause yielding of biofilms grown from lab strains with different patterns of polysaccharide production. (B) For all lab-strain biofilms except Δ*mucA*, the contribution to breaking energy that comes from elastic (stored) energy contributions is ~10× greater than the contribution that comes from viscous (dissipated) energy contributions.

**Figure S10**. Schematics of rheology measurements. (A and B) The rheometer tool (two parallel, black plates) applies a mechanical shear to the biofilm (green). (A) shows the unstrained state; (B) shows displacement of the top plate to impose a shear strain ε = Δx/y. (C) For elastic solids, the slope of the linear regime of the stress-strain curve gives the material’s elastic modulus G′. The end of the linear regime marks the yield point, which correlates with a yield strain and yield stress. (D) Using our strain-sweep rheology measurements, we determined biofilm yield as the point of intersection between a linear fit to the plateau elastic modulus G′ and a power-law fit to the region of decreasing G′ with strain.

**Figure S11**. Time sweep of WT to evaluate drying with and without solvent trap. Elastic modulus G′ was measured for 10 minutes at constant strain and frequency. (A) Without the solvent trap, the stiffness of the biofilm increases with time as a result of water loss. (B) With the solvent trap, biofilm stiffness stays constant in time. The solvent trap alleviates the stiffness increase due to drying. (C) Image of the rheometer tool with the solvent trap in place.

**References for Supplementary Information**

1. Mahenthiralingam, E., M. Campbell, and D. Speert, *Nonmotility and phagocytic resistance of Pseudomonas aeruginosa isolates from chronically colonized patients with cystic fibrosis.* Infection and Immunity, 1994. **62**: p. 596-605.

2. Huse, H., T. Kwon, J. Zlosnik, D. Speert, E. Marcotte, and M. Whiteley, *Parallel Evolution in Pseudomonas aeruginosa over 39,000 Generations In Vivo.* mBio, 2010. **1**: p. e00199-10.

3. Huse, H., T. Kwon, J. Zlosnik, D. Speert, E. Marcotte, and M. Whiteley, *Pseudomonas aeruginosa Enhances Production of a Non-Alginate Exopolysaccharide during Long-Term Colonization of the Cystic Fibrosis Lung.* PloS ONE, 2013. **8**: p. e82621.

4. Holloway, B., *Genetic recombination in Pseudomonas aeruginosa.* Journal of General Microbiology, 1955. **13**: p. 572-581.

5. Starkey, M., J. Hickman, L. Ma, N. Zhang, S. De Long, A. Hinz, S. Palacios, C. Manoil, M. Kirisits, T. Starner, D. Wozniak, C. Harwood, and M. Parsek, *Pseudomonas aeruginosa rugose small-colony variants have adaptations that likely promote persistence in the cystic fibrosis lung.* Journal of Bacteriology, 2009. **191**: p. 3492-3503.

6. Borlee, B., A. Goldman, K. Murakami, R. Samudrala, D. Wozniak, and M. Parsek, *Pseudomonas aeruginosa uses a cyclic-di-GMP-regulated adhesin to reinforce the biofilm extracellular matrix.* Molecular Microbiology, 2010. **75**: p. 827-842.

7. Kirisits, M., L. Prost, M. Starkey, and M. Parsek, *Characterization of colony morphology variants isolated from Pseudomonas aeruginosa biofilms.* Applied and Environmental Microbiology, 2005. **71**: p. 4809-4821.

8. Jacobs, M., A. Alwood, I. Thaipisuttikul, D. Spencer, E. Hougen, S. Ernst, O. Will, R. Kaul, C. Raymond, R. Levy, L. Chun-Rong, D. Guenterner, D. Bovee, M. Olson, and C. Manoil, *Comprehensive transposon mutant library of Pseudomonas aeruginosa.* Proceedings of the National Academy of Sciences of the USA, 2003. **100**: p. 14339–14344.

9. Hutchison, J., C. Rodesney, K. Kaushik, H. Le, D. Hurwitz, Y. Irie, and V. Gordon, *Single-cell control of initial spatial structure in biofilm development using laser trapping.* Langmuir, 2014. **30**: p. 4522-4530.
