# Supplementary figures and images for: Evolutionary adaptations of biofilms infecting cystic fibrosis lungs promote mechanical toughness by adjusting polysaccharide production
Source: NPJ Biofilms Microbiomes. 2017 Jan 23;3:1. doi: 10.1038/s41522-016-0007-9 (PMC5445605; doi:10.1038/s41522-016-0007-9)

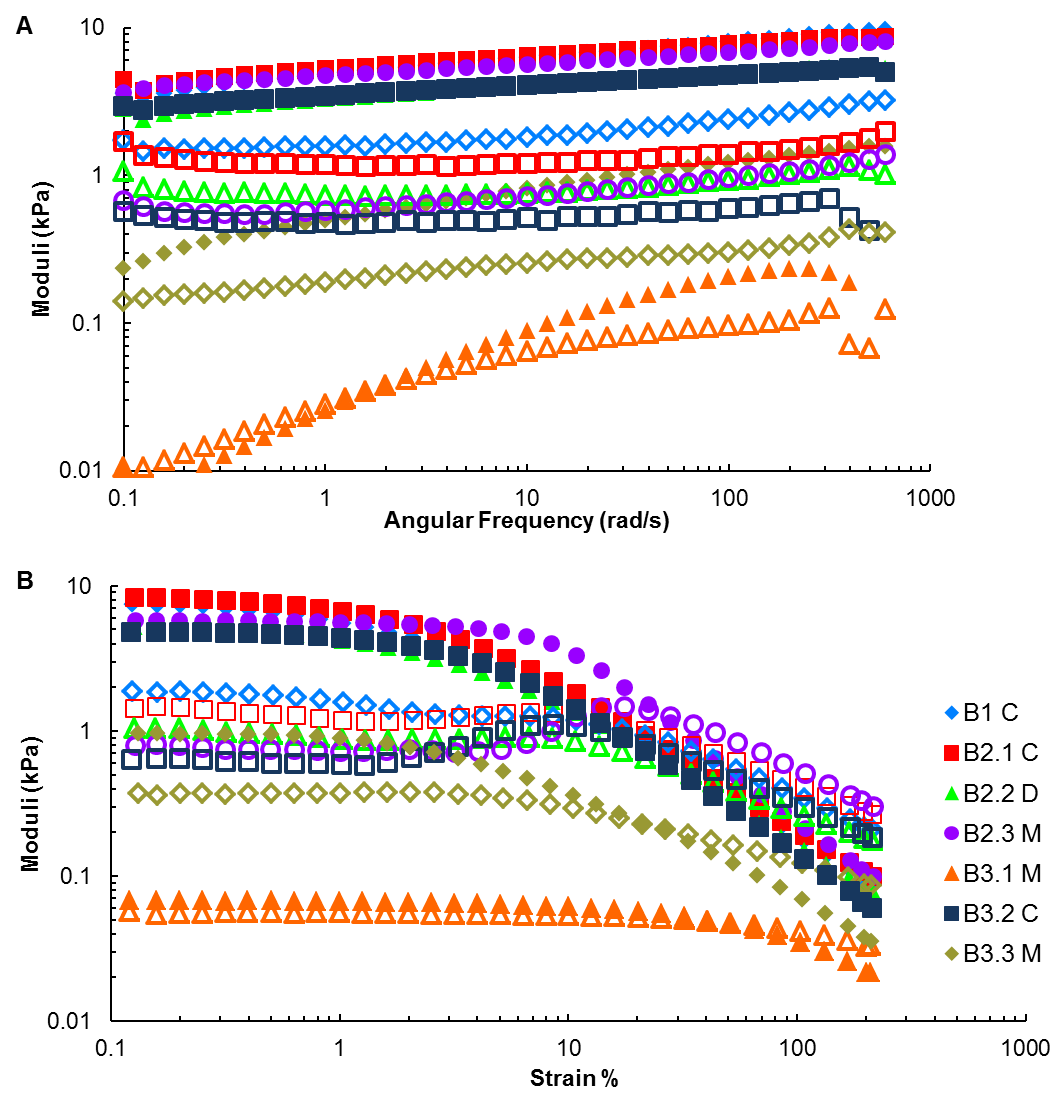

Supplement: Supplementary file 2 — Figure S1 [file 41522_2016_7_MOESM2_ESM.tif]

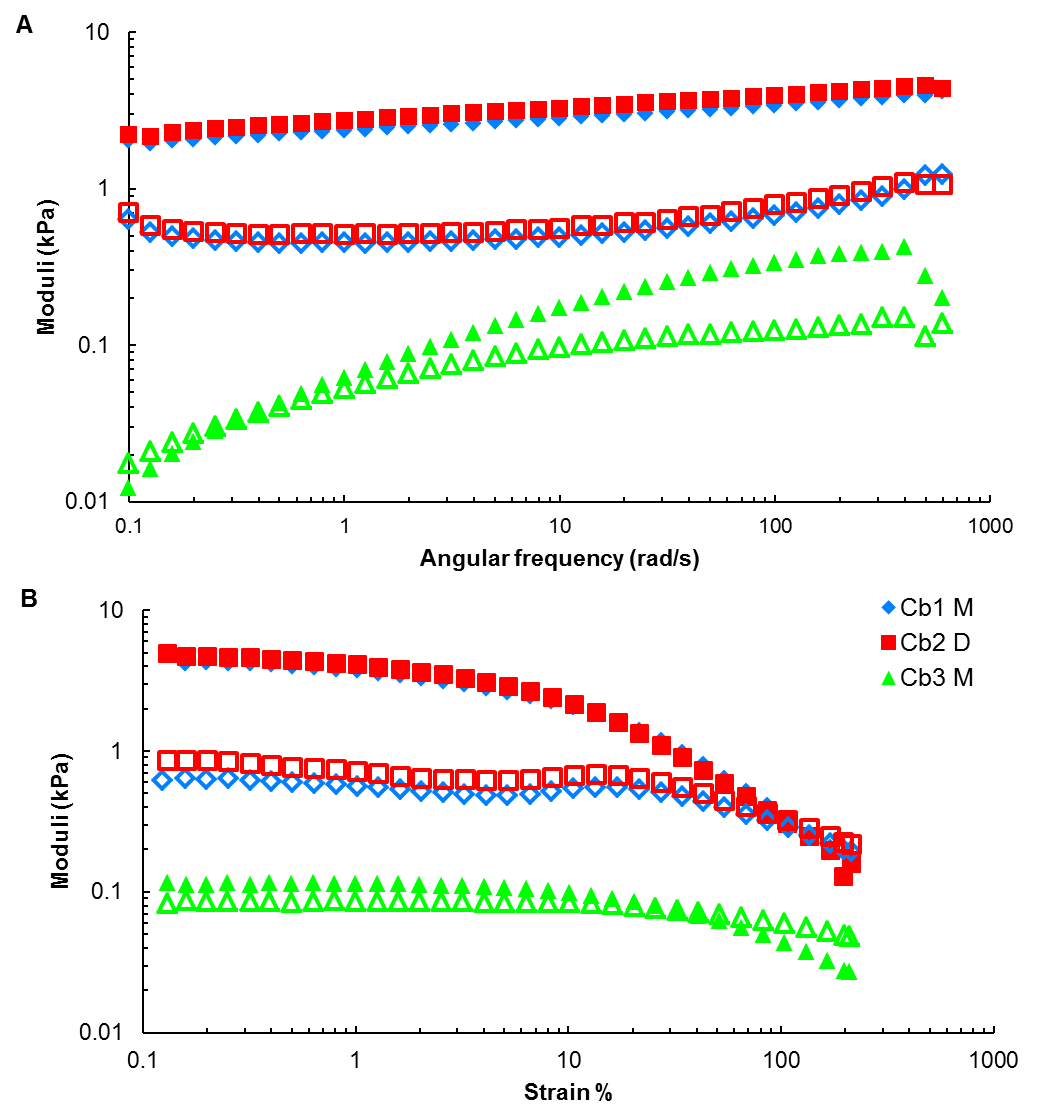

Supplement: Supplementary file 3 — Figure S2 [file 41522_2016_7_MOESM3_ESM.tif]

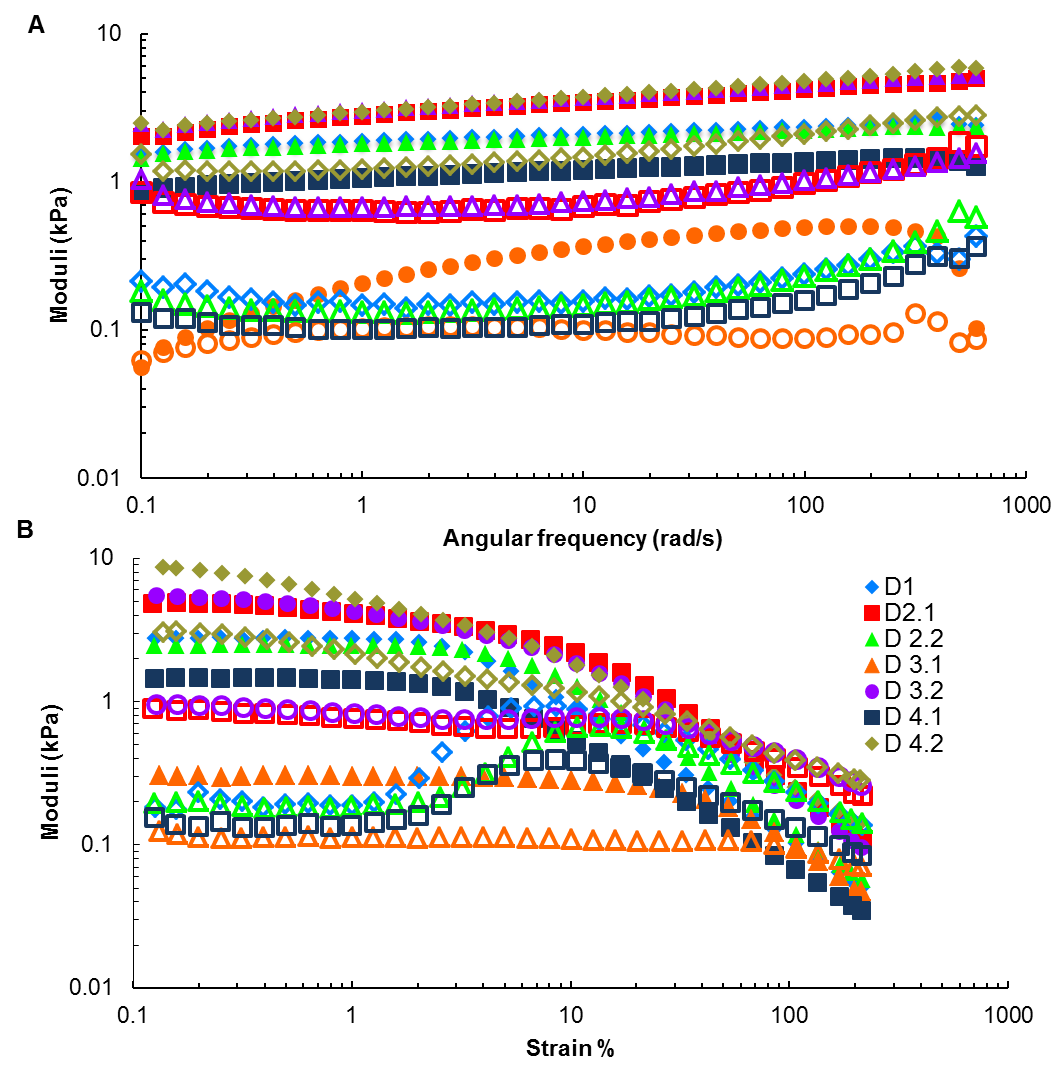

Supplement: Supplementary file 4 — Figure S3 [file 41522_2016_7_MOESM4_ESM.tif]

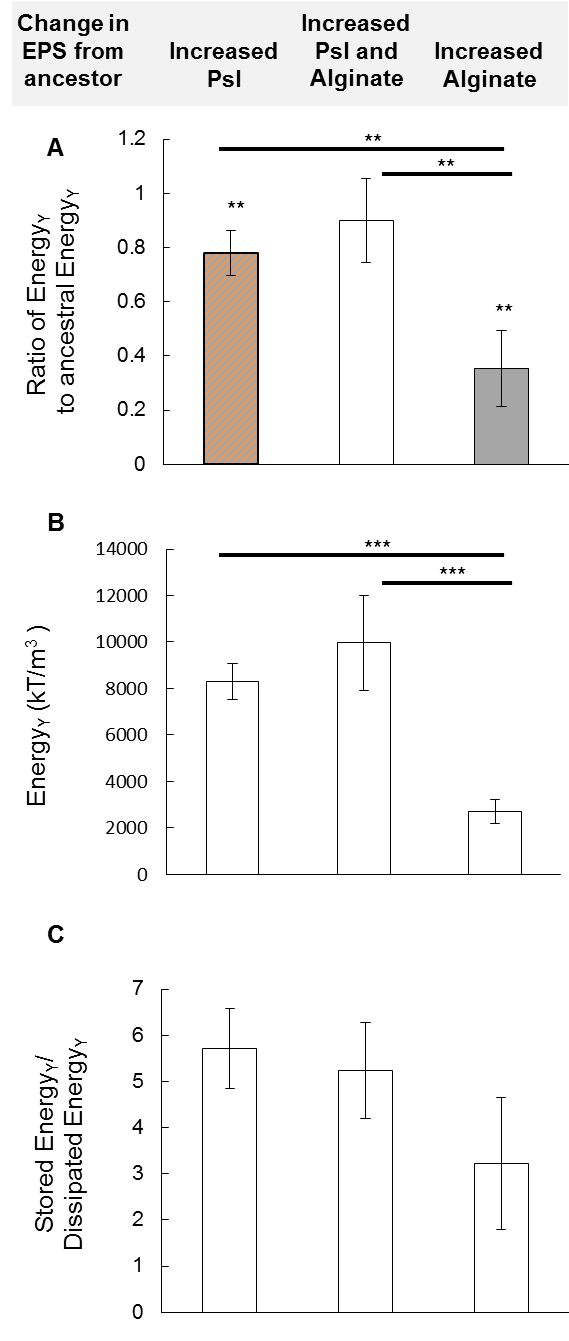

Supplement: Supplementary file 5 — Figure S4 [file 41522_2016_7_MOESM5_ESM.tif]

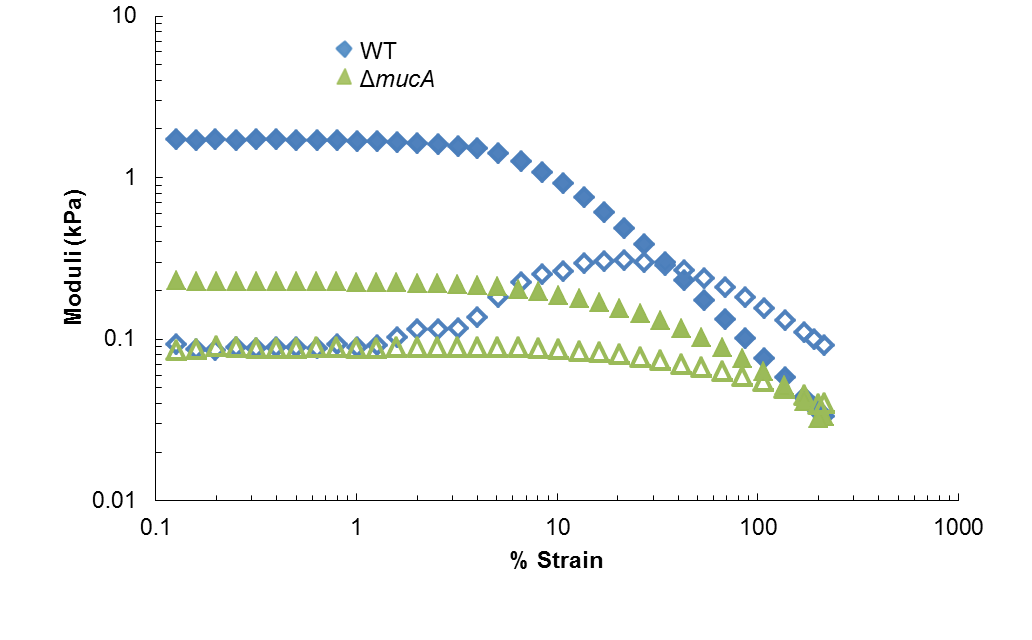

Supplement: Supplementary file 6 — Figure S5 [file 41522_2016_7_MOESM6_ESM.tif]

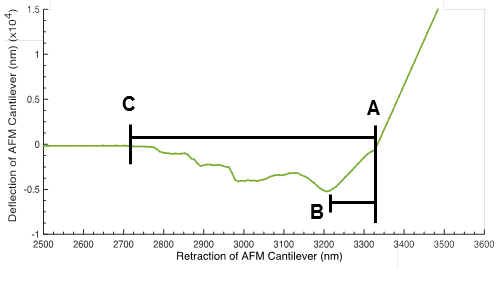

Supplement: Supplementary file 7 — Figure S6 [file 41522_2016_7_MOESM7_ESM.tif]

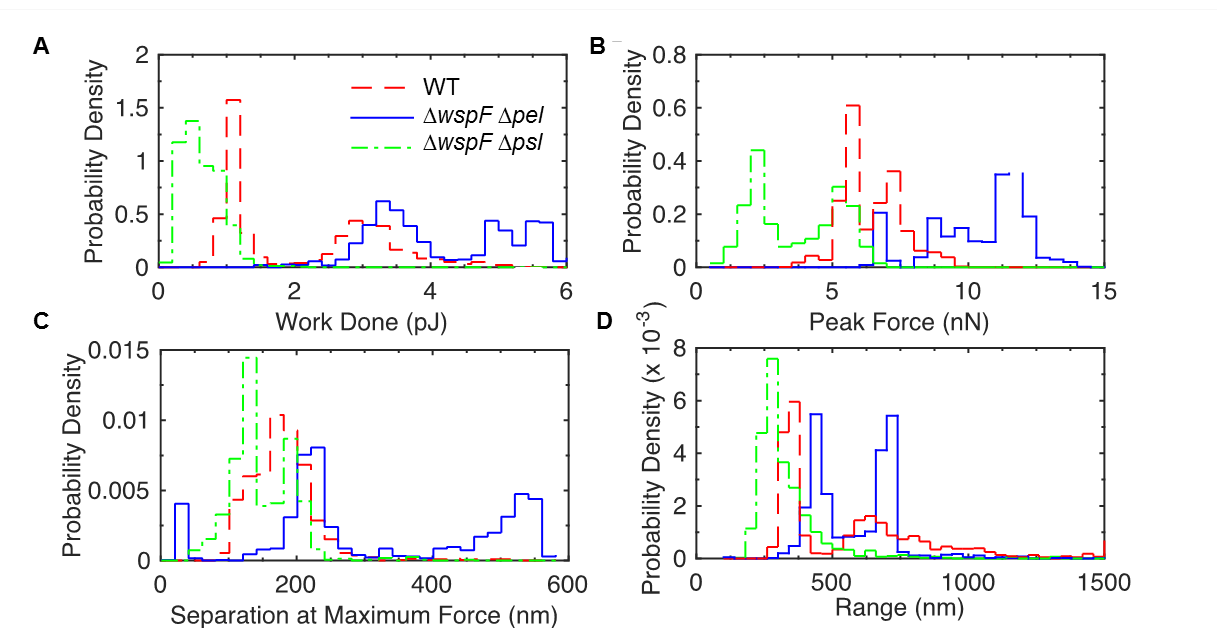

Supplement: Supplementary file 8 — Figure S7 [file 41522_2016_7_MOESM8_ESM.tif]

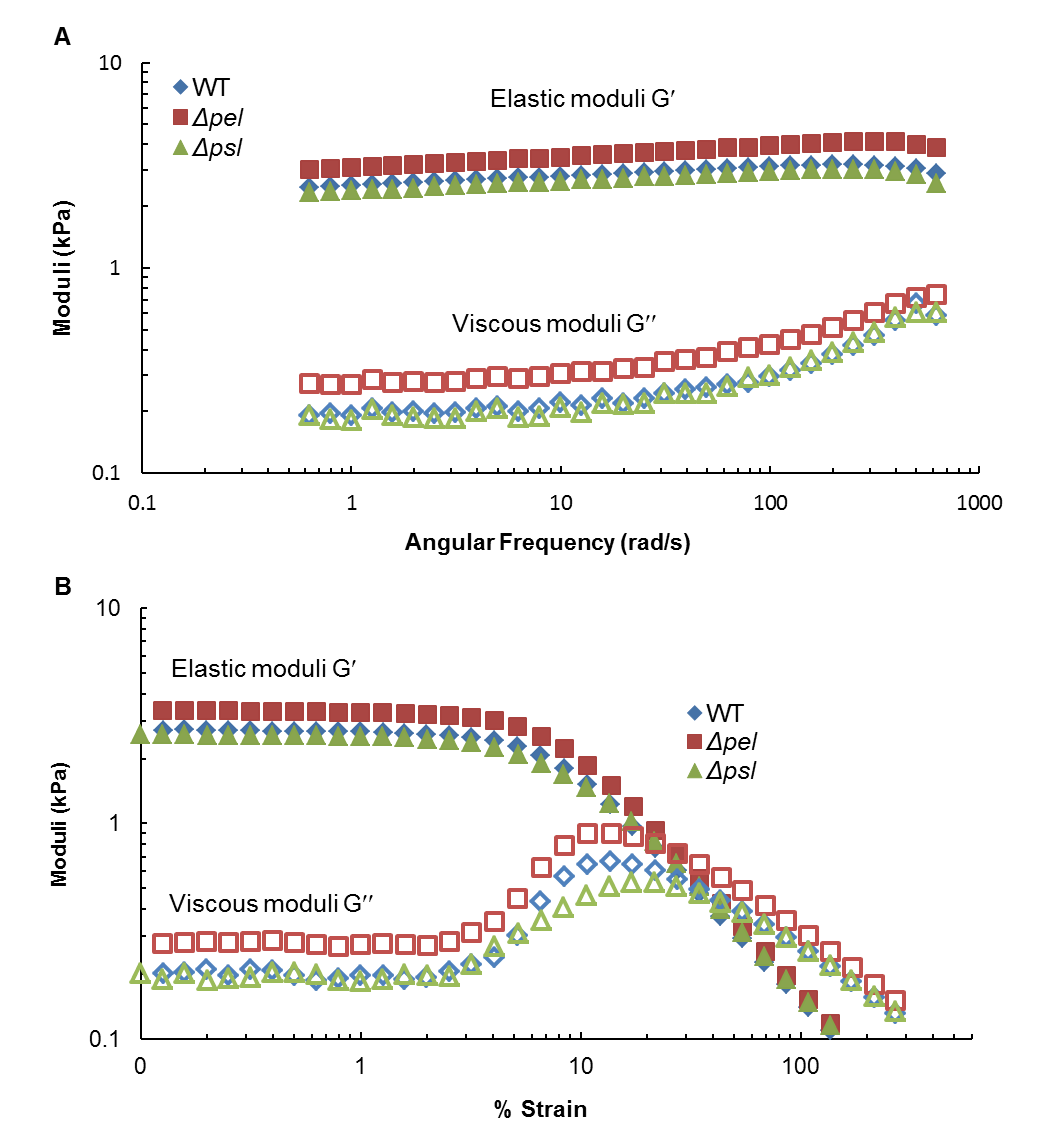

Supplement: Supplementary file 9 — Figure S8 [file 41522_2016_7_MOESM9_ESM.tif]

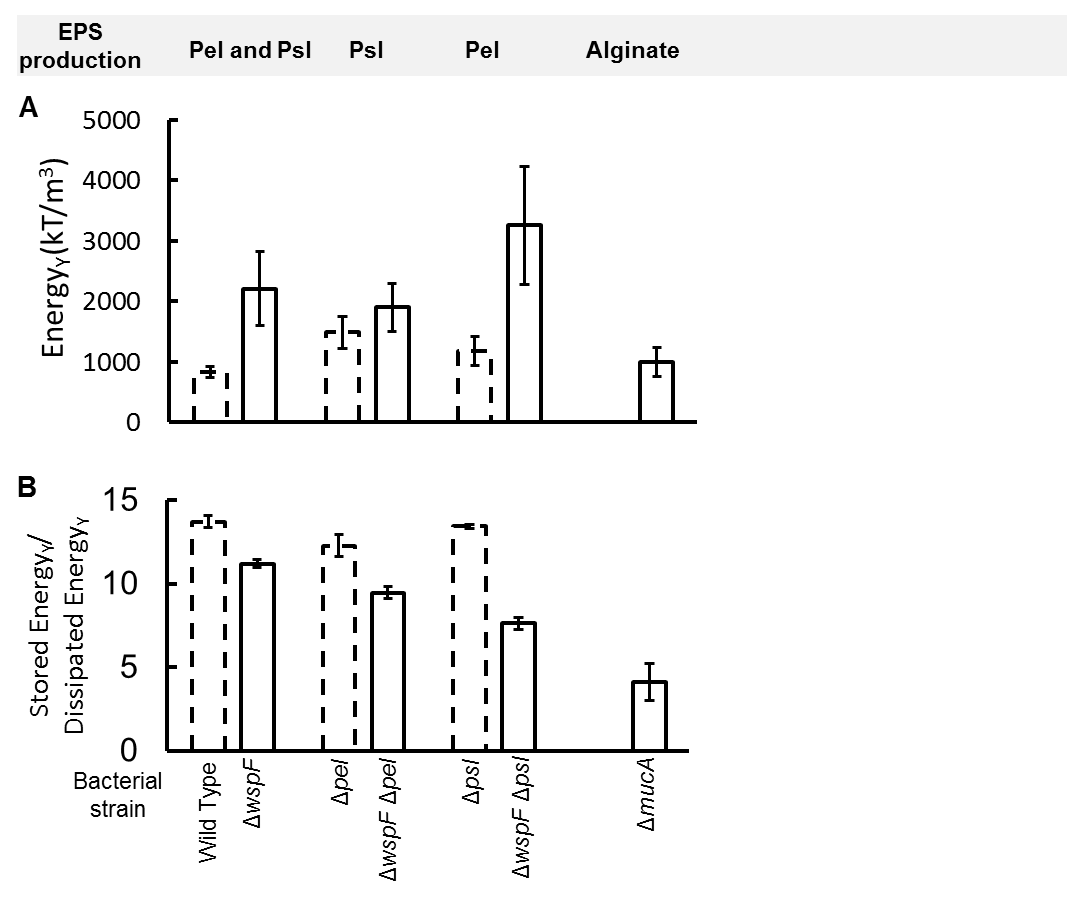

Supplement: Supplementary file 10 — Figure S9 [file 41522_2016_7_MOESM10_ESM.tif]

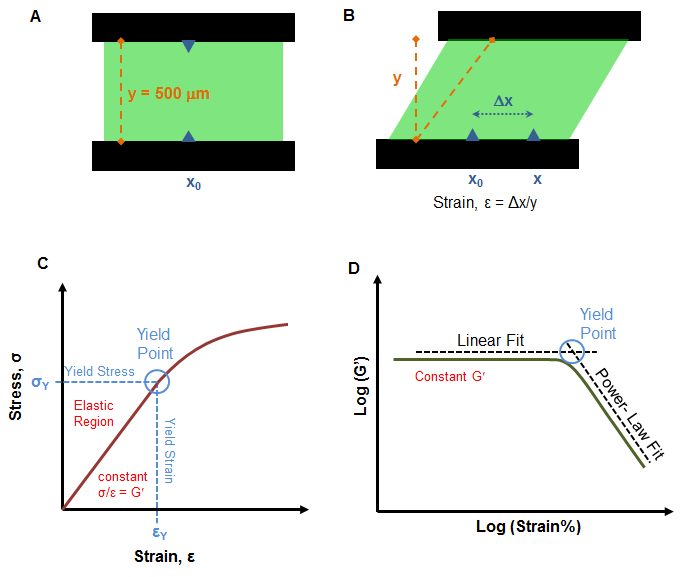

Supplement: Supplementary file 11 — Figure S10 [file 41522_2016_7_MOESM11_ESM.tif]

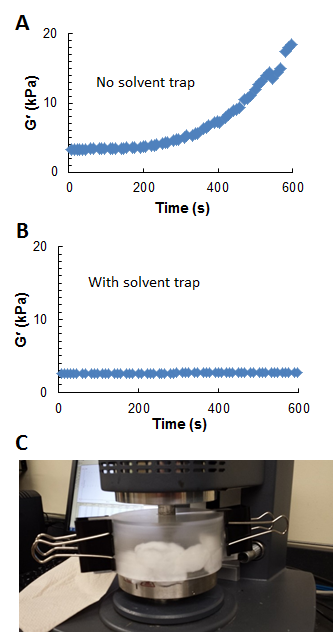

Supplement: Supplementary file 12 — Figure S11 [file 41522_2016_7_MOESM12_ESM.tif]
